# Genetic Diversity and Population Structure of *Didymella* *rabiei* Affecting Chickpea in Ethiopia

**DOI:** 10.3390/jof7100820

**Published:** 2021-09-30

**Authors:** Gezahegne Getaneh, Tadele Tefera, Fikre Lemessa, Seid Ahmed, Tarekegn Fite, Jandouwe Villinger

**Affiliations:** 1 Ambo Agricultural Research Center, Ethiopian Institute of Agricultural Research, Addis Ababa P.O. Box 2003, Ethiopia; 2 International Centre of Insect Physiology and Ecology (*icipe*), Addis Ababa P.O. Box 5689, Ethiopia; ttefera@icipe.org (T.T.); tfduressa@gmail.com (T.F.); 3 International Centre of Insect Physiology and Ecology (*icipe*), Nairobi P.O. Box 30772-00100, Kenya; jandouwe@icipe.org; 4 Department of Horticulture and Plant Science, Jimma University, Jimma P.O. Box 307, Ethiopia; fikre.lemessa@gmail.com; 5 ICARDA International Center of Agricultural Research, Rue Hafiane Cherkaoui, Agdal, Rabat P.O. Box 6299, Morocco; s.a.kemal@cgiar.org; 6 School of Plant Sciences, College of Agriculture and Environmental Sciences, Haramaya University, Dire Dawa P.O. Box 138, Ethiopia

**Keywords:** Ascochyta blight, chickpea blight, *Didymella* *rabiei*, diversity, population structure, ITS

## Abstract

Ascochyta blight, also known as chickpea blight, which is caused by the fungal pathogen, *Didymella* *rabiei*, is an important disease affecting chickpea (*Cicer arietinum* L.) in many countries. We studied the genetic diversity and population structure of 96 *D.* *rabiei* isolates collected from three geographic populations in Ethiopia using simple sequence repeat (SSR) markers. We confirmed the genetic identity of 89 of the *D. rabiei* isolates by sequencing their rRNA internal transcribed spacer region genes. The chickpea blight pathogen isolates were genetically diverse, with a total of 51 alleles identified across 6 polymorphic SSR loci, which varied from 3 to 18 (average 8.5) alleles per SSR marker. The observed heterozygosity and expected heterozygosity ranged from 0.01 to 0.92 and 0.19 to 0.86, respectively. The mean polymorphic information content value of the *D. rabiei* populations was 0.58, with a mean gene diversity of 0.61 among loci. Gene flow (Nm = number of migrants) for the three populations of *D. rabiei* isolates ranged from 1.51 to 24.10 (average 6.2) migrants/cluster. However, the genetic variation between the *D. rabiei* populations was small (8%), with most of the variation occurring within populations (92%). Principal component analysis to visualize genetic variation showed that the *D. rabiei* isolates obtained from most of the chickpea samples formed roughly three groups on a two-dimensional coordinate plane. Similarly, the clustering of individuals into populations based on multi-locus genotypes (using Clumpak) grouped isolates into three clusters but with individual isolate admixtures. Hence, no clear geographic origin-based structuring of populations could be identified. To our knowledge, this is the first report of *D. rabiei* diversity in Ethiopia. Virulence studies should be conducted to develop chickpea varieties that are resistant to more aggressive pathogen populations.

## 1. Introduction

Chickpea (*Cicer arietinum* L.) is the most important grain legume in the world after the common bean (*Phaseolus vulgaris*) and pea (*Pisum sativum*) [1]. Ethiopia is the largest chickpea producing country in Africa, accounting for approximately 46% of total production [1]. Ethiopia mainly produces desi-type chickpea with limited acreage of the kabuli type [2], and the crop covers 15.2% of the area that is allocated for pulse crops cultivated in Ethiopia [2]. The crop is grown for home consumption and income generation (local and foreign markets) and to restore fertility as part of a crop rotation with major cereal crops such as wheat and tef [3].

Ascochyta blight of chickpea (*Ascochyta rabiei* (Pass) Labr. (Teleomorph: *Didymella rabiei* (Kovachevski) var. Arx.), also known as chickpea blight, is the most important yield- and quality-limiting factor worldwide [4,5]. The disease has been reported in over 40 countries in Asia (Bangladesh, China, India, Iran, Iraq, Israel, Jordan, Lebanon, Pakistan, Syria, and Turkey), Africa (Algeria, Egypt, Ethiopia, Kenya, Libya, Morocco, Sudan, Tanzania, and Tunisia), Europe (Cyprus, Bulgaria, France, Greece, Hungary, Italy, Portugal, Romania, Spain, and Ukraine), North America (Canada and the USA), and South America (Argentina, Colombia, and Mexico) as well as Australia [6,7,8]. Under favorable environmental conditions, the disease can cause yield losses of up to 100% on susceptible chickpea crops [8,9,10]. The rapidly increasing trend of chickpea production in many countries is limited by chickpea blight epidemics, and currently, approximately 95% of the chickpea growing areas worldwide are prone to chickpea blight epidemics [11,12,13].

Pathogens evolve and change their virulence and aggressiveness in response to sexual reproduction and gene flow, with selection pressure from resistant cultivars. The population of *D. rabiei* is known for its variability in virulence/aggressiveness, and many popular cultivars of chickpea have been taken out of production, and no chickpea germplasm has complete resistance to chickpea blight; rather, cultivars quantitively differ in their resistance or susceptibility [8]. The phytotoxin produced by the pathogen initially cause gray areas on the leaves, stems, and pods that rapidly turn into brown lesions with dark borders. As the disease progresses, small circular brown and black dots (pycnidia) develop in the center of these lesions and are arranged in concentric cercles, which is the most important diagnostic characteristic of the disease. In severe cases, the lesions are enlarged, girdle the stem, and the entire plant dries up suddenly, with small patches of brown dead plants observed in the field [14,15,16,17,18]. The disease can be managed through the application of fungicides and through the use of resistant cultivars [19]. However, the emergence of new virulent pathogen populations is becoming a challenge in order to develop and release high-yielding and blight-resistant cultivars [8]. The genetic diversity of *D. rabiei* populations has been studied using different hosts and molecular markers [20]. Morphologically, the characterization of fungal genetic diversity is difficult, as it has limited characters and is mostly affected by environmental conditions. Hence, many molecular techniques such as restriction fragment length polymorphism (RFLP), rRNA internal transcribed spacer (ITS), random amplified polymorphic DNA (RAPD), amplified fragment length polymorphism (AFLP), simple sequence repeat (SSR), and single nucleotide polymorphism (SNP) have been used to characterize *D. rabiei* genetic diversity [18,21]. Microsatellites are the most widely used molecular markers for population diversity studies of *D. rabiei* due its high level of polymorphism, even distribution over the genome, co-dominance, relative ease of detection, transferability among related species, and abundance of DNA sequences in eukaryotic genomes [21,22,23]. In this study, we used microsatellite simple sequence repeat (SSR) markers, which were developed by Geistlinger et al. [24]. SSR markers are widely used in genetic diversity studies, and approximately 25 different microsatellite motifs have been reported for the *D. rabiei* genome [24,25,26,27,28,29].

In Ethiopia, chickpea blight was first reported in 1969 [30]. Currently, the disease is distributed across all of the major chickpea production areas of the country, but the population genetic diversity of *D. rabiei* is not yet known in Ethiopia. The objective of this study was to determine the genetic diversity and population structure of Ethiopian isolates of *D. rabiei* using molecular markers.

## 2. Materials and Methods

### 2.1. Sample Collection, Pathogen Isolation and DNA Extraction

**Figure 1:**
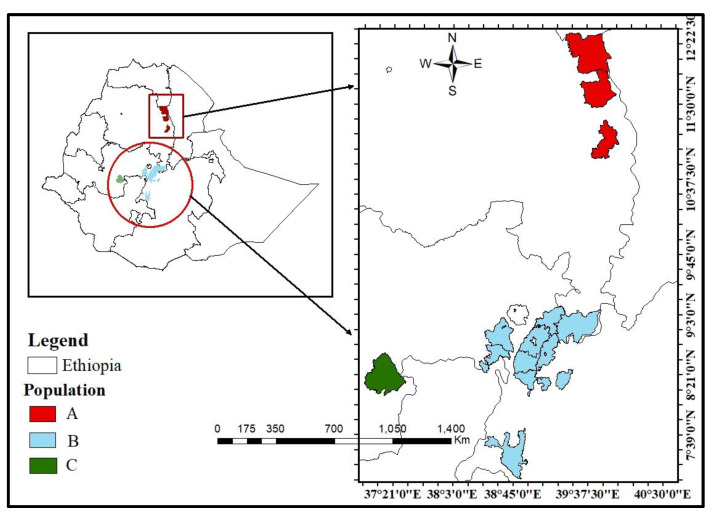
Isolate collection of *D. rabiei* from different chickpea growing agro-ecologies of Ethiopia. (A = northern population; B = central population; C = southern population).

Isolates of chickpea plant parted infected by *D. rabiei* were randomly collected using a stratified sampling technique, where the sampling was done from farmer fields and Ethiopian and Amhara regional agricultural research stations and were grouped into three geographic locations (central region = Pop-A, northern region = Pop-B, and southern region = Pop-C) in the major chickpea growing area (Figure 1). The collections were completed in the 2016/17 and 2017/18 cropping seasons (August to January). A total of 96 isolates were collected from infected chickpea plant parts (leaves, seed coats, and stems) with typical symptoms of necrotic lesions with circular blights. The blight-infected chickpea samples were cut into pieces (approximately 1 cm of stem or single leaflets), surface disinfected with 1.5% concentrated house bleach (NaOCl) for 1 min and rinsed with sterile distilled water. Surface-disinfected samples were placed on 2% water agar medium (HiMedia Laboratories Pvt. Ltd., Mumbai, India) and were incubated at room temperature (~25 °C) for 48–72 h. Following the production of pycnidium, the infected samples were removed, and small cubes of water agar with discharged conidia were excised and transferred to potato dextrose agar (PDA) (HiMedia Laboratories Pvt. Ltd., Mumbai, India). Cultures were incubated at 25 °C for 5 to 7 days. When pure colonies of *D. rabiei* were obtained, isolates were single spored by spreading them in 0.1 mL of distilled sterile water on the surface of PDA media using glass spreaders and were incubated at 25 °C for 10 to 15 days and were stored at −20 °C for further work. For DNA extraction, isolates were first cultivated on PDA at 20 °C for 10 to 15 days. Mycelium was scraped from the surface of the plates and was used to initiate cultures in 250 mL flasks containing 50 mL of liquid 2-yeast extract glucose (YEG) medium (2 g/L yeast extract, 10 g/L glucose). After 5–6 days on a rotary shaker at 150 rpm and 23 °C, mycelia from the flasks were lyophilized in 9 cm Petri dishes. The mycelia of each isolate were finely ground using liquid nitrogen, and total genomic DNA was extracted following the manufacturer’s protocols for the Plant Kit^®^ (Bioline Ltd., Indore, India).

### 2.2. Pathogen Sequencing

The rRNA ITS1-5.8S-ITS4 gene region of the *D. rabiei* isolates was amplified using two primers, ITS1 (5′-TCC GTA GGT GAA CCT GCG G-3′) and ITS4 (5′-TCC TCC GCT TAT TGA TAT GC-3′), as described by White et al. [31]. The primers were mixed in equal concentrations in a single PCR. PCRs were conducted in 10-µL volumes containing 1 µL of genomic DNA, 5 µL of PCR master mix HotStarTaq^®^ (QIAGEN Group, Venlo, Netherlands), and 0.5 µL of each primer. Cycling conditions consisted of an initial denaturation at 95 °C for 15 min followed by 35 cycles at 94 °C for 30 s, 56 °C for 1 min, at 72 °C for 1 min, and a final extension at 72 °C for 7 min. Amplified PCR products were visualized on a 1% agarose gel stained with ethidium bromide (4 ng/mL) and were purified with a QuikClean Gel Extraction Kit (GenScript Corporation, Piscataway, NJ, USA) according to the manufacturer’s protocols. A 100 base-pair (bp) DNA ladder (Invitrogen, Waltham, MA, USA) was run in the outer lanes of the gel as a size standard. Representative isolates (*n* = 89) of the amplified ITS region of rRNAs were sequenced.

### 2.3. SSR Amplification

Six SSR primers developed by Geistlinger et al. [24] were used for the genetic diversity analysis of *D. rabiei* (Table 1). The *D. rabiei* isolates were amplified using the forward (and reverse primers tailed a with universal M13(-21) sequence (5′-TGT AAA ACG ACG GCC AGT-3′) along with a complementary FAM-labeled M13(-21) universal primer (FAM-5′-TGT AAA ACG ACG GCC AGT-3′). PCRs were conducted in 10-µL volumes containing 1 µL of genomic DNA, 5 µL of PCR master mix HotStarTaq^®^ (QIAGEN Group), 0.5 µL each of forward and reverse primer as well as the FAM-labelled M13(-21) universal primer. Amplifications were performed in a GeneAmp PCR System 9700 thermocycler (Applied Biosystems, Foster City, CA, USA) with the following cycling conditions: 95°C for 15 min followed by 35 cycles at 94 °C for 30 s, 56 °C for 1 min, 72°C for 1 min, and a final extension at 72 °C for 7 min. GeneScan 500 LIZ dye size standard (Thermo Fisher scientific, Waltham, Massachusetts, USA) was used according to the manufacturer’s recommendations, and PCR without template DNA was used as control. Fragment analysis was outsourced from the University of Illinois, USA (https://unicorn.biotech.illinois.edu/, accessed on 18 August 2020).

### 2.4. Data Analyses

#### 2.4.1. Internal Transcribed Spacer Region

For confirmation of *D. rabiei* infection, homologous ITS sequences identified using the Basic Local Alignment Search Tool (BLAST) in GenBank (of the National Centre for Biotechnology Information) were aligned to those obtained from 89 isolates using Geneious Prime 2020.2.3 software.

#### 2.4.2. SSR Polymorphism and Genetic Diversity

Peak identification and fragment sizing of SSR loci were performed using Geneious software with the default settings. Then, the allele size data at each locus were exported to Excel for statistical analyses. Polymorphisms were calculated for basic parameters of locus-based diversity indices: major allele frequency (MAF), number of alleles (NA), gene diversity (GD), and polymorphic information content (PIC) using PowerMarker ver. 3.25 [32]. The number of effective alleles per locus, number of different alleles per locus, number of private alleles, observed heterozygosity, expected heterozygosity, unbiased heterozygosity, and Shannon’s information index were estimated for each population using GenAlEx version 6.503 [33]. The amount of polymorphism was estimated, and gene diversity was calculated for each selected SSR marker across 96 isolates according to the Nei diversity index [34].

#### 2.4.3. Analysis of Molecular Variance

To estimate the variance components of the populations and the distribution of gene diversity, analysis of molecular variance (AMOVA) was estimated using GenAlEx molecular software. Population differentiation (PhiPT) of the whole population and pairwise PhiPT among all pairs of populations were determined, and significance was tested based on 1000 bootstraps. An unweighted neighbor-joining dendrogram was constructed. The AMOVA based on codominant SSR loci was estimated using GenAlEx version 6.503 [33].

#### 2.4.4. Analyses of Allelic Patterns

The equations used are as follows: Na = number of different alleles with a frequency ≥ 5%; Ne = number of effective alleles (1/(Sum pi^2^)); *I* = Shannon’s information index (−1 × Sum (pi × Ln (pi))); *N*o. of private alleles = number of alleles unique to a single population; He = expected heterozygosity = (1 − Sum pi^2^); and uHe = unbiased expected heterozygosity = ((2*N*/(2*N* − 1)) × He).

#### 2.4.5. Cluster Analysis, Principal Component Analysis, and Population Genetic Structure

The phylogenetic relationships of 96 isolates were constructed using the unweighted neighbor-joining approach with 1000 bootstrap replicates using DARwin molecular software version 6.0.010 (http://darwin. cirad.fr, accessed on 29 September 2021. Principal coordinate analysis (PCoA) was completed using GenAlEx software to show the genetic differentiation pattern of the populations. Population structure was inferred using a Bayesian model-based clustering algorithm designed in STRUCTURE version 2.3.4. To determine the most appropriate number of populations (*K*), a burn-in period of 25,000 was used in each run, and data were collected over 100,000 Markov chain Monte Carlo replications from *K* = 1 to *K* = 10. This procedure groups individuals into populations and estimates the proportion of membership in each population of individuals [23,35]. The *K* value was determined by the log probability of data (Ln P(D)) based on the rate of change in LnP(D) between successive *K* [36]. The optimum *K* value was predicted following the simulation method of Evanno et al. [37] using the web-based software Structure Harvester version 0.6.92 [36]. The PCoA was computed to show the genetic variation patterns in the populations of *D. rabiei* isolates.

## 3. Results

### 3.1. Pathogen Identity

High quality ITS nucleotide sequences (502 bp) were obtained from 89 isolates, all of which shared 100% identity with the *D. rabiei* sequences in GenBank (NR_136126_TYPEmaterial, MH861656_Syria, KT962077_India, MH861657_Syria, MH244158_Turkey, KM977755_Pakistan, KY465495_China, EU595358_Hungary, and many others). A single consensus nucleotide sequence was obtained and was deposited in GenBank (Accession number OK161019).

### 3.2. SSR Polymorphism and Gene Diversity

Six (ArH02T, ArA03T, ArA06T, ArA08T, ArH05T, and ArR01D) polymorphic and informative SSR markers were used for genetic diversity analyses. The overall allele size ranged from 164 to 435 bp (Table 1). Across the six polymorphic SSR markers, total of 51 alleles were detected in the 96 *D. rabiei* isolates that were studied, and the number of alleles per locus ranged from 3 (ArA08T, ArR01D) to 18 (ArH02T), with an average of 8.5 per locus (Table 2).

The observed heterozygosity and expected heterozygosity ranged from 0.01(ArR01D) to 0.92 (ArH02T) and 0.19 (ArA08T ArR01D) to 0.86 (ArH02), respectively. The maximum observed heterozygosity was detected at the ArH02T locus with a value of 0.92, and the minimum was at the ArR01D locus with a value of 0.01; the expected heterozygosity (He) ranged from 0.19 to 0.86, with an average of 0.56. The effective number of alleles (Ne) ranged from 1.24 to 7.23.

Many loci were polymorphic, and the mean PIC was 0.58, with a range between 0.29 (ArR01D) and 0.86 (ArH02T). The highest (0.82) major allele frequency was observed at the ArR01D locus, whereas the lowest was observed at the ArH02T locus. The mean gene diversity was 0.61, whereas the highest (0.86) was observed at the ArH05T locus, and the lowest (0.31) was observed at the ArR01D locus. The ArH02T and ArH05T loci provided the most information in this diversity study, with a higher (18 and 15, respectively) allele number, and the fewest alleles were found at the ArR01D and ArA08T loci (three alleles each). The number of genotypes was higher at the ArH05T locus (18), but this number was lower at ArH08T and ArR01D, which had a value of four. Pairwise cluster estimates of gene flow (Nm) for the three populations ranged from 1.51 to 24.10 (average 6.2) migrants per cluster, and a low *F*_ST_ value (0.039) was obtained according to Nei’s genetic distance analysis. With the exception of ArA08T, all of the evaluated markers significantly deviated from the Hardy-Weinberg equilibrium.

The mean values for the number of different alleles (Na) and the effective number of alleles (Ne) across all six loci of the overall population were 4.22 and 2.68, respectively (Table 3), and the mean values for the overall population in I, He, and uHe were 0.91, 0.47, and 0.50, respectively. Pop-A (*I* = 1.179, He = 0.564, and uHe = 0.568) showed higher diversity than Pop B and Pop-C. The percentage of polymorphic loci per population ranged from 66.67% (Pop-C) to 100% (Pop-A), with an average of 83.33%. The number of private alleles was higher in Pop-C (0.67) than it was in the other populations.

### 3.3. Population Genetic differentiation and Gene Flow

The AMOVA based on the codominant SSR loci showed that the percentage of genetic variation between the populations was small (8%). Most of the observed variation occurred within populations (92%) (Table 4). In addition, the gene flow was (Nm) was 2.7. Therefore, genetic variation mainly exists within the *D. rabiei* populations. Hence, most of the within-population variation is due to the heterozygosity of the individuals within each population. In addition, population differentiation, the PhiPT of the total populations, and the pairwise PhiPT among all of the pairs of populations were determined and were found to be significant (Table 4).

### 3.4. Population Genetic Diversity Analysis

The genetic diversity analysis of the three *D. rabiei* populations revealed that the effective number of alleles (Ne) and expected heterozygosity (He) across all six loci varied from 2.28 to 3.36 and 0.40 to 0.56, respectively (Table 5). A higher mean number of different alleles per locus (Na) value (6.5) was observed in the central population (Pop-A), while a lower value (2.5 and 3.67) was recorded in the southern (Pop-B) and northern population (Pop-C), respectively. Similarly, unbiased gene diversity (uHe) was higher in the central population (uHe = 0.568). The percent of polymorphic loci varied from 66.67% (Pop-C) to 100% (Pop-A), with an average of 83.33% (Table 5). Private alleles were detected in all of the populations, with most of them occurring in the central population (Pop-A) (Table 5). The PhiPT and gene flow (Nm) were 0.072 (*p* < 0.001) and 3.73, respectively (Table 4).

### 3.5. Cluster Analysis, Principal Component Analysis, and Population Genetic Structure

**Figure 2:**
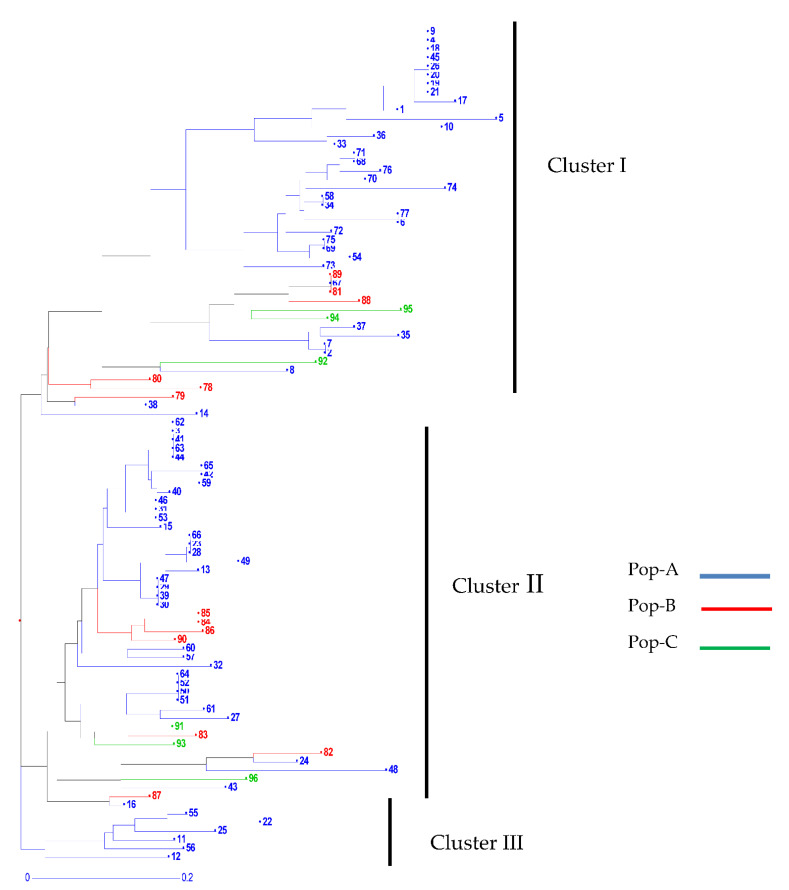
Phylogenetic relationship among 96 isolates constructed using the unweighted neighbor-joining approach with 1000 bootstrap replicates. (The populations are color-coded as follows: blue = Pop-A; red = Pop-B; and green = Pop-C).

The unweighted neighbor-joining dendrogram grouped the 96 isolates of the three populations into three major clusters (Cluster-I, Cluster-II, and Cluster-III) with some isolates being present in different clusters (Figure 2). The overall topology of the dendrogram indicated the presence of three clades in the studied *D. rabiei* isolates. Of the 96 isolates, 45, 45, and 6 isolates were grouped in clusters I, II, and III, respectively. Several subclades were observed for the populations, indicating genetic variability within and among the isolates in each population.

**Figure 3:**
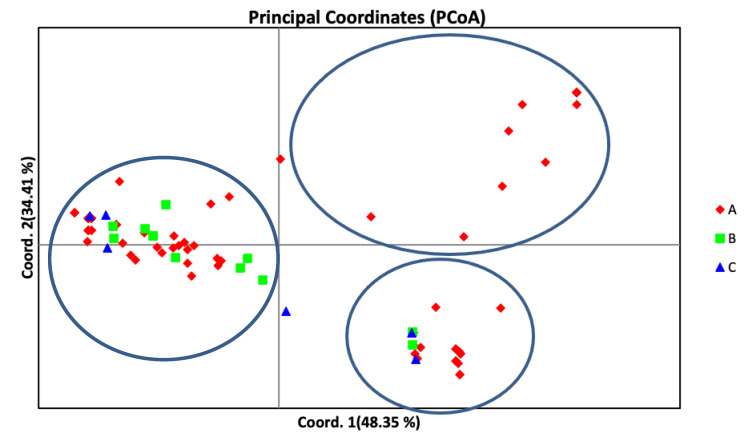
Principal-coordinate analysis showing the diversity of *D. rabiei* isolates clustered in the three subpopulations collected from three regions (A = Pop-A; B = Pop-B; C = Pop-C). The circles represent the grouping of the isolates.

**Figure 4:**
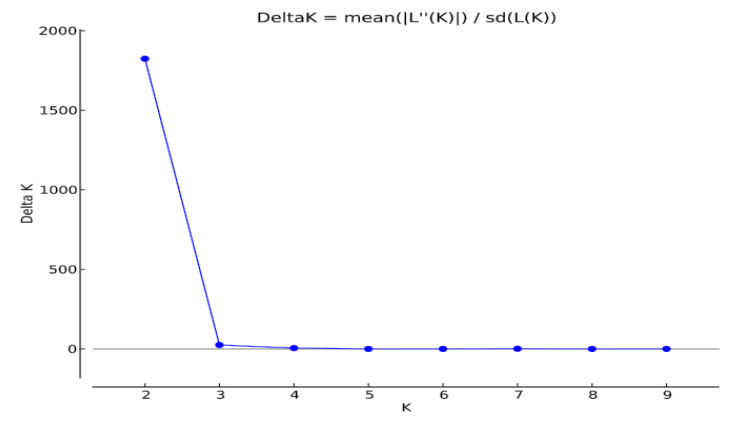
Log probability of the data (LnP(D)) for structure estimation of the number of subgroups from *K*-values ranging from 1 to 10 by ΔK values (gray line = initial modal value).

**Figure 5:**
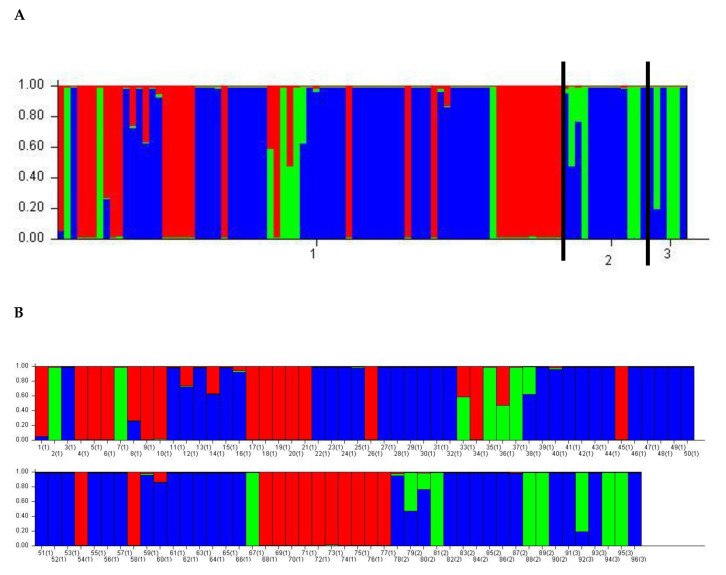
(**A**) Bayesian model-based estimation of population structure (*K* = 2) for 96 *D. rabiei* isolates into three groups (blue, red, and green), and each vertical line represents one individual, and each population is divided by a black line (numbered as 1,2,3). Each color shows the genetic composition that is assigned to a different genetic cluster (**B**) Population structure analysis (blue, Pop-A; red, Pop-B; green, Pop-C; (the isolates that did not share more than 70% of ancestry were considered admixtures). The lengths of the colored segments on the *Y*-axis show the estimated membership proportions of each isolate in the group (coefficient of membership), and the numbers on the *X*-axis refer to the isolate numbers.

Principal component analysis (PCoA), which was used to identify genetic variation pattern among *D. rabiei* isolates, showed that the isolates were roughly grouped into three major groups, and the first axis explained 34.41% of the total variation, the second axis explained 48.35% of the variation, explaining 82.76% of the total variation (Figure 3). However, the STRUCTURE outputs predicted delta *K* = 2 to be the most likely number of clusters (Figure 4). Clumpak (bar plot) clustering of individuals into populations based on multi-locus genotypes grouped the isolates into three clusters with an admixture of isolates; hence, there was no clear geographic origin-based structuring of the populations (Figure 5).

## 4. Discussion

This study confirms that *D. rabiei* was the causative agent of chickpea blight in Ethiopia and that there are no sequence differences in the ITS1-5.8S-ITS2 regions of the isolates. This result could be due to the origin of the source pathogen and the involvement of humans in pathogen movement via seeds, contributing to geographic spread [6]. Ethiopia, with poor phytosanitary services, imports germplasm every year for breeding purposes [38]; this germplasm could possibly be infected.

The six SSR loci of *D. rabiei* isolates were polymorphic, with a mean PIC of 0.58, ranging between 0.86 and 0.29. Three loci were highly informative (0.5 < PIC > 0.25), and three were reasonably informative (0.5 < PIC > 0.25) [39]. The PIC indicated an estimate of the discriminatory power of the loci using the numbers and relative frequencies of the alleles [40].

Though different levels of genetic diversity of *D. rabiei* have been reported from other countries, we detected relatively high (H = 0.61) genetic variability in Ethiopian populations, which were comparable to the highest levels of diversity that were observed in Iran (0.79) and Turkey (0.69) [41,42]. Tunisia, Canada, the USA, and Syria showed diversities of 0.55, 0.38, 0.36, and 0.32, respectively [43,44,45]. Low genetic diversity has been reported in Australia (0.066 and 0.094) [46,47]. Genetic diversity is affected by the age of a population, population size, selection, mutation, population gene flow, and genetic recombination.

The average estimated gene flow (Nm) (the movement of genes into or out of a population) among the populations was 3.73, and the relatively low F_ST_ value (0.063) between the *D. rabiei* populations could be due to gene flow among the populations. Gene flow could have resulted from anthropogenic activities such as the exchange of infected seeds for planting among framers, the introduction of infected s breeding material seeds, genetic drift, and migration [48]. Informal seed exchange (farmer to farmer) without certification is a common practice in Ethiopia. According to McDonald [48], when the gene flow statistic (Nm) is < 1 and no genotypes are shared between populations, populations are considered isolated (not exchanging genes or genotypes), suggesting that quarantine measures have been effective. Similarly, the average F_ST_ across all of the loci (*F*_ST_ = 0.03) and the pairwise F_ST_ for all of the population pairs (highest value = 0.07) were generally low to moderate based on Wright (1943), which indicates that genetic differentiation among populations can be considered high, as the value of F_ST_ was greater than 0.25 [49]. A separation of the isolates into geographically distinct subpopulations was not observed, and this could be due to pathogen movement by infected seeds in different parts of the country [4,50].

Host resistance to chickpea blight in chickpea has not been durable in some countries because of the high pathogen virulence variability in popular chickpea cultivars [13]. Sexual reproduction and selection pressure imposed by growing few varieties over large areas are the most likely causes for the emergence of resistant pathogen populations [51,52]. In Ethiopia, we recently found both mating types (MAT1-1 and MAT1-2) with a ratio of 1:9, respectively, with which sexual reproduction can most likely occur, leading to higher genotypic variably [53]. The role of sexual reproduction may not be an important factor to bring changes in Ethiopian *D. rabiei* populations as the low temperature required for sexual reproduction does not exist in the country [54]. The genetic analysis of the pathogen is important for efficient disease management strategies. The aggressiveness of *D. rabiei,* which is controlled by a specific gene, was identified from different chickpea-producing countries with different levels of disease severity [15,46,55,56]

The *D. rabiei* isolates from the different geographic locations in Ethiopia did not show clear genetic differences (admixture). A higher mean number of different alleles per locus (Na) was observed in the central population (Pop-A), but this could be due to the broadest geographical range of this population. The relative genetic distances among populations did not completely correlate with the geographical distances of their sampling locations. Genetic diversity may not indicate pathogenic aggressiveness, but it is important to test the aggressiveness of pathogens on a set of hosts with different levels of resistance [42,56,57,58]. Intensive diversity with pathogenic virulence studies should be addressed regularly to support chickpea resistance breeding programs in Ethiopia.

Based on this preliminary study, we suggest additional studies with multiple isolates of both local and global strains to evaluate the long-term stability of this characteristic in haplotype classification. The Ethiopian isolates clearly showed genetic differences within and between collection areas, and the isolates collected from research stations and farmer fields did not show significant differences; the isolates from geographically distinct populations indicate that admixture has occurred. Most of the genetic variability was attributable to diversity within locations rather than between locations.

## 5. Conclusions

Chickpea blight, caused by the fungal *Ascochyta* teleomorph *D. rabiei*, is the most important chickpea disease in most chickpea growing areas around the world. We found genetically diverse Ascochyta blight pathogens in Ethiopia, where chickpea production has increased every growing season, even in areas where chickpea has not been previously grown, as producers earn reasonable prices for this cash crop. Unless the pest burden can be managed, there will be great economic losses for producers and the country in case of epidemics. Understanding of the genetic diversity of this pathogen will help in the development of improved disease management strategies for the sustainable production of chickpea as a source of food and income. The genetic variability of Ethiopian isolates may result from the genetic recombination of the pathogen or may be acquired through seeds imported for variety trials. Genetic diversity still does not necessarily indicate pathogenic aggressiveness. Hence, the virulence of this pathogen should be studied on appropriate hosts or on chickpea blight differential lines, and phytosanitary rules should be applied when moving seeds within the country or from overseas.

## Figures and Tables

**Table 1:** Characteristics of six polymorphic microsatellite markers in the current population genetic diversity study of *D. rabiei.*

Locus	Forward Primer Sequences (5′ to 3′)	Reverse Primer Sequences (5′ to 3′)	Expected Allele Size	Repeat Motifs
ArH02T	CTGTATAGCGTTACTGTGTG	TCCATCCGTCTTGACATCCG	273–411	GAA and GTA
ArA03T	TAGGTGGCTAAATCTGTAGG	CAGCAATGGCAACGAGCACG	285–435	GAA
ArA06T	CTCGAAACACATTCCTGTGC	GGTAGAAACGACGAATAGGG	164–188	CAACAC and CAC
ArA08T	CAGAGGGGAATTGTTGTTC	ACGACGAGGATGAGGACTTC	264–267	CTTCCT and CTT
ArH05T	CATTGTGGCATCTGACATCAC	TGGATGGGAGGTTTTTGGTA	213–285	CTT
ArR01D	CAGAGGGGAGTCACAAGTATC	GAGTTACAGCTGCAAGACATTC	181–213	GTGTGTGG

**Table 2:** Molecular characteristics of the six SSR microsatellite loci based on three *D. rabiei* populations.

Marker	MAF	Gn	Na	Ne	*I*	Gd	Ho	He	uHe	PIC	Nm	*F*	HWE		
													ChiSq	Prob (*P*)	Sign.
ArH02T	0.21	16	18	7.23	2.29	0.87	0.92	0.86	0.87	0.86	2.40	-0.11	993.99	0.000	***
ArA03T	0.45	14	8	3.38	1.47	0.73	0.19	0.70	0.71	0.70	3.52	0.72	272.773	0.000	***
ArA06T	0.53	5	4	2.11	0.83	0.53	0.17	0.53	0.53	0.42	24.10	0.68	60.328	0.000	***
ArA08T	0.77	4	3	1.24	0.34	0.38	0.17	0.19	0.20	0.35	6.15	0.00	0.001	0.978	ns
ArH05T	0.27	18	15	6.66	2.18	0.86	0.55	0.85	0.86	0.85	1.51	0.31	641.142	0.000	***
ArR01D	0.82	4	3	1.31	0.40	0.31	0.01	0.24	0.24	0.29	1.81	0.95	82.756	0.000	***
Mean	0.51	10.17	8.5	3.66	1.25	0.61	0.33	0.56	0.57	0.58	6.58	0.43			

MAF (major allele frequency), Gn (number of genotypes), Na (number of alleles), Ne (number of effective alleles), *I* (Shannon’s information index), Gd (gene diversity), Ho (observed heterozygosity), He (expected heterozygosity), PIC (polymorphic information content), uHe (unbiased expected heterozygosity), Nm (Number of migrant (gene migrant)), F(fixation index), HWE (Hardy–Weinberg equilibrium), probability significance level; ns = not significant, *** *p* < 0.001.

**Table 3:** Genetic diversity estimates for three populations of *D. rabiei* based on six microsatellite (SSR) loci.

Population	*N*	Na	Na Freq. ≥ 5%	Ne	*I*	No. Private Alleles	He	uHe	% of Polymorphic Loci
Pop-A	77	6.50	3.33	3.36	1.18	3.33	0.56	0.57	100.00
Pop-B	13	3.67	2.67	2.41	0.86	0.33	0.46	0.48	83.33
Pop-C	6	2.50	2.50	2.28	0.69	0.67	0.40	0.44	66.67
Mean		4.2	2.83	2.68	0.91	1.44	0.47	0.50	83.33

*N* = sample size; Na = mean number of different alleles; Na (Freq ≥ 5%) = number of different alleles with a frequency ≥ 5%; Ne = number of effective alleles; *I* = Shannon’s information index; No. Private Alleles = number of alleles unique to a single population; He = expected heterozygosity; uHe = unbiased expected heterozygosity.

**Table 4:** Analyses of molecular variance (AMOVA) based on six SSR loci and estimates of gene migration (Nm) among and within three *D. rabiei* populations.

Source	df	SS	MS	Est. Var.	%	Stat	Value	*p*
Among Pops	2	26.11	13.10	0.49	8%	PhiPT	0.085	0.05
Within Pops	93	488.13	5.25	5.25	92%	Nm	2.7	
Total	95	514.24		5.74	100%			

df = degree of freedom; SS = sum of squares; MS = Mean square; Est.Var= estimated variance; % = variation percent; Stat.= calculated statistics.

**Table 5:** Mean diversity indices of six *D. rabiei* SSR loci for three populations in Ethiopia.

Population	N	Na	Ne	Na Freq. ≥ 5%	I	No. Private Alleles	Ho	He	uHe	% of Polymorphic Loci
Pop-A	73.00	6.50	3.36	3.33	1.18	3.33	0.34	0.56	0.57	100.00
Pop-B	12.33	3.67	2.41	2.67	0.86	0.33	0.46	0.46	0.48	83.33
Pop-C	5.33	2.50	2.28	2.50	0.69	0.67	0.28	0.40	0.44	66.67
Mean										83.33

*N* = sample size; Na = mean number of different alleles; Ne = number of effective alleles; Na (Freq ≥ 5%) = number of different alleles with a frequency ≥ 5%; *I* = Shannon’s information index; No. Private Alleles = number of alleles unique to a single population; Ho = observed heterozygosity; He = expected heterozygosity; uHe = unbiased expected heterozygosity.

## Data Availability

The study didn’t report any data.
